# Efficacy and Safety of ramucirumab Combined With Nab‐paclitaxel, Lobaplatin, and S‐1 in Conversion Therapy for Unresectable Advanced Gastric Cancer: Preliminary Results of Conversion Therapy Cohort of RNPLS‐01 Trial

**DOI:** 10.1002/mco2.70958

**Published:** 2026-11-03

**Authors:** Juan Wang, Wei Wang, Guanghui Xu, Shushang Liu, Longxing Jiao, Shu Wang, Chaosheng Peng, Yuxuan Ma, Xin Liu, Huade Huo, Haolin Li, Xiaohua Li, Jianjun Yang

**Affiliations:** ^1^ Department of Digestive Surgery Xijing Hospital of Digestive Diseases Fourth Military Medical University Xi'an China; ^2^ Department of General Surgery Yulin Hospital Affiliated to the First Affiliated Hospital of Xi'an Jiaotong University Yulin China; ^3^ Department of Pharmaceutics and Pharmacy Administration School of Pharmacy Air Force Medical University Xi'an Xi'an China; ^4^ Xi'an Medical University Xi'an China

**Keywords:** conversion therapy, lobaplatin, nab‐paclitaxel, ramucirumab, S‐1, unresectable advanced gastric cancer

## Abstract

This single‐center randomized controlled trial assessed the efficacy and safety of the ramucirumab plus nab‐paclitaxel, lobaplatin, and S‐1(RNPLS) regimen for conversion therapy in unresectable advanced gastric cancer. Seventy patients were equally assigned to receive either ramucirumab, nab‐paclitaxel, lobaplatin, and S‐1(RNPLS) therapy or nab‐paclitaxel, lobaplatin, and S‐1(NPLS) chemotherapy alone. Primary endpoints included pathological complete response (pCR) and secondary endpoints encompassed R0 resection rate, objective response rate (ORR), the disease control rate (DCR), progression‐free survival (PFS), overall survival (OS), duration of response (DOR), and adverse events (AE). Although the difference in pCR rate was statistically insignificant between the two groups, the RNPLS group achieved significantly higher ORR, DCR, and R0 resection rate. Moreover, the RNPLS regimen yielded prolonged PFS, longer duration of response, and promising preliminary OS. While hypertension occurred more frequently in the RNPLS group, the incidence of grade ≥3 AEs was low and comparable in both cohorts. This exploratory single‐center randomized trial showed preliminary favorable efficacy of RNPLS regimen for conversion therapy in unresectable advanced gastric cancer with manageable toxicity.

**Trial Registration:**

ClinicalTrials.gov: NCT06169410

## Graphical abstract

This study enrolled 70 HER2‐negative, unresectable stage III/IV gastric/gastroesophageal junction adenocarcinoma patients with over 6‐month survival, divided into RNPLS and NPLS groups (*n* = 35 each). Both groups received 21‐day‐cycle chemotherapy with lobaplatin, nab‐paclitaxel, and S‐1, while RNPLS additionally used ramucirumab. RNPLS yielded significantly better pCR, ORR, DCR, R0 resection rate, DOR, PFS, and OS. It had higher hypertension incidence, yet presented rare grade ≥3 adverse events.

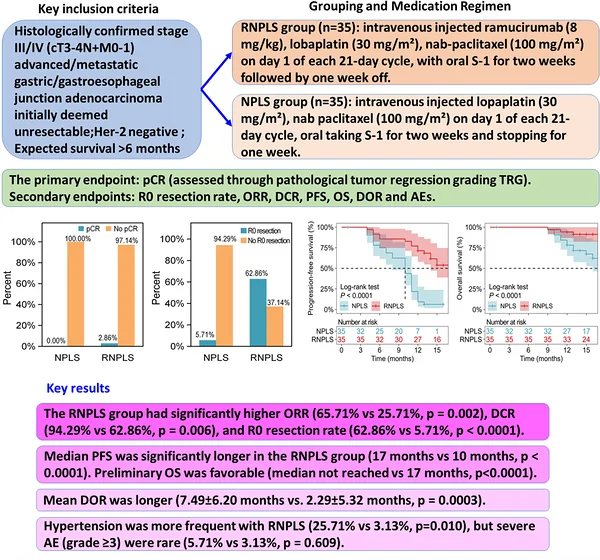

## 1 Introduction

Gastric cancer (GC) persists as a therapeutically intractable global malignancy, ranking fourth in incidence yet second in cancer‐related mortality due to persistent limitations in treatment efficacy [1]. Despite advances in systemic therapies, the prognosis for patients with unresectable advanced GC remains dismal. The primary therapeutic challenge stems from the disease being inoperable at diagnosis in most GC patients in China, leaving systemic chemotherapy as the mainstay of treatment.

First‐line regimens for unresectable advanced GC, typically platinum (cisplatin or oxaliplatin) combined with a fluoropyrimidine (5‐fluorouracil [5‐FU] or capecitabine), achieve a median overall survival (OS) of only 9–11 months. Existing second‐line therapies, such as EGFR inhibitors (cetuximab), mTOR inhibitors (everolimus), or bevacizumab (which failed in phase III trials like AVAGAST), offer only marginal survival gains and highlight a critical unmet therapeutic need [2, 3, 4]. First‐line regimens face limitations due to intrinsic or acquired chemoresistance, tumor heterogeneity, commonly late‐stage diagnosis, and significant toxicity profiles—all of which limit dose intensity and treatment duration. Oxaliplatin, a cornerstone in advanced GC treatment per NCCN guidelines, is favored over cisplatin due to its lower renal and gastrointestinal toxicity. However, oxaliplatin frequently causes cumulative sensory neuropathy (grade ≤2: 41.6%). To improve therapeutic effect, lobaplatin has emerged as a significant therapeutic option for advanced unresectable GC, particularly in China, where clinical studies have demonstrated its efficacy and manageable toxicity profile [5]. As a third‐generation platinum analog, lobaplatin is often combined with fluoropyrimidines like S‐1 or tegafur, whose combination has shown efficacy in Asian populations, suggesting their compatibility in multimodal regimens [6]. In addition, albumin‐bound paclitaxel (nab‐paclitaxel) has emerged as an effective therapeutic option for advanced unresectable GC, demonstrating significant efficacy in both first‐line and subsequent‐line settings [7]. Compared to solvent‐based cytotoxic drugs such as taxanes and 5‐FU, albumin‐bound paclitaxel improves tolerability while maintaining efficacy. Recent clinical studies in GC patients have demonstrated that nab‐paclitaxel significantly improved OS versus solvent‐based paclitaxel (mOS: 9.6 vs. 8.8 months; HR 0.92, 95% CI 0.73–1.15) and ORR (28% vs. 16%) with lower neurotoxicity (grade ≥2: 17% vs. 29%) and comparable hematologic toxicity [8]. Meanwhile, a phase II study of nab‐paclitaxel in combination with ramucirumab in patients with previously treated advanced GC found that the ORR was 54.8% and the DCR was 92.9%. The median PFS was 7.6 months [9]. Therefore, given the clinical challenges in advanced GC management, particularly balancing efficacy and tolerability, there has been a growing shift in practice toward replacing the cisplatin/5‐FU backbone with a multimodal regimen combining lobaplatin, nab‐paclitaxel, and S‐1, implying the promising efficacy and a more favorable toxicity profile in Asian populations

Over the past decade, immune checkpoint inhibitors (ICIs targeting PD‐1/PD‐L1) have drastically reshaped the first‐line therapeutic landscape of HER2‐negative unresectable advanced GC. Multiple pivotal global phase III randomized trials including CheckMate‐649, ATTRACTION‐4, KEYNOTE‐859, ORIENT‐16, GEMSTONE‐303, and RATIONALE‐305 have established PD‐1 inhibitor plus platinum‐fluoropyrimidine chemotherapy as the category 1 recommended first‐line standard by NCCN, CSCO, and ESMO guidelines, with significantly improved OS and PFS especially in patients with PD‐L1 CPS ≥1 [10, 11, 12, 13, 14, 15]. For HER2‐positive advanced disease, pembrolizumab added to trastuzumab plus chemotherapy has been approved for CPS ≥1 population following KEYNOTE‐811 positive findings [16]. Nevertheless, substantial unmet needs persist under current chemo‐immunotherapy framework. Nearly half of patients with low/negative PD‐L1 expression fail to derive meaningful survival benefit from standard ICI‐combination treatment [10, 17]. Immune‐related adverse events (AEs) restrict long‐term immunotherapy delivery in a subset of patients and conversion‐to‐resectable rate remains suboptimal (∼30%–40%) with routine chemo‐immunotherapy for initially unresectable stage III/IV GC, highlighting urgent requirement to develop alternative conversion regimens beyond conventional chemo‐immunotherapy backbone [18, 19, 20, 21, 22, 23].

Recently, clinical evidence showed that vascular endothelial growth factor (VEGF)‐targeted agents represent a significant advancement in managing advanced unresectable GC, primarily through inhibition of tumor angiogenesis—a critical process in which tumors develop new blood vessels to support their growth and metastasis [24]. Ramucirumab, a recombinant anti‐VEGFR2 IgG1 monoclonal antibody targeting VEGFR‐2, stands as the first globally approved angiogenesis inhibitor for second‐line GC treatment, capable of binds VEGFR2 to block activation by VEGF‐A, VEGF‐C, and VEGF‐D ligands and downstream pro‐angiogenic cascades, thereby suppressing endothelial cell proliferation, migration, and neovascularization [25]. This mechanism not only starves tumors but also normalizes aberrant tumor vasculature, reducing interstitial pressure and improving chemotherapeutic drug delivery [25]. The landmark RAINBOW trial demonstrated ramucirumab's survival benefit. Combined with paclitaxel, it achieved a median OS of 9.6 months versus 7.4 months for paclitaxel alone (HR 0.81, 19% risk reduction), alongside improved median progression‐free survival (PFS: 4.4 vs. 2.9 months) and objective response rate (ORR: 28% vs. 16%) [26]. Asian subgroups, including Chinese patients, derived consistent benefits (OS: 8.71 vs. 7.92 months) [27]. These outcomes highlight ramucirumab's role in conversion therapy. Beyond standalone second‐line application of ramucirumab, accumulating preclinical and emerging real‐world data confirm that VEGFR2 blockade‐mediated vascular normalization ameliorates tumor immunosuppressive microenvironment and facilitates intratumoral lymphocyte infiltration, laying biological foundation for future combinatorial strategy of ramucirumab‐based multi‐chemo plus PD‐1 inhibitors in conversion setting, which represents a promising direction to overcome immunotherapy resistance in PD‐L1‐low GC population [26, 28, 29, 30, 31, 32, 33, 34, 35, 36, 37, 38, 39].

Currently, chemoimmunotherapy is the mainstream clinical treatment, yet it yields suboptimal conversion outcomes in patients with low PD‐L1 expression and those intolerant to immune checkpoint inhibitors. Current studies have suggested the promising efficiency of ramucirumab, nab‐paclitaxel, lobaplatin, and S‐1 in advanced unresectable GC. However, the role of the combination of ramucirumab, nab‐paclitaxel, lobaplatin, and S‐1 in the conversion therapy of advanced unresectable GC remains to be verified. Our previous study has shown that ramucirumab combined with nab‐paclitaxel, lobaplatin, and S‐1 significantly enhances the pCR rate and ORR in the neoadjuvant therapy of locally advanced GC patients [40]. Due to the fact that conversion therapy typically employs neoadjuvant therapy regimens, this study hypothesizes that combined VEGFR‐2 inhibition, enhanced taxane delivery, and synergistically cytotoxic anti‐tumor effect may improve resectability rates while maintaining tolerability. Here, we present the preliminary efficacy and safety results from the conversion therapy cohort of the RNPLS‐01 trial.

## 2 Results

### 2.1 Clinicopathological Characteristics of Enrolled Patients

A total of 70 eligible patients were enrolled between December 6, 2023 and July 16, 2024, comprising the intention‐to‐treat population. Table 1 provides detailed baseline demographic and clinical characteristics of both study groups. The two groups exhibited comparable baseline demographics and clinical profiles (Table 1). Figure 1 presents the CONSORT flow diagram detailing patient enrollment and allocation procedures.

**TABLE 1:** Comparison of baseline demographic and clinicopathologic characteristics of the enrolled patients.

Clinicopathologic characteristics	RNPLS group (*n* = 35)	NPLS group (*n* = 35)	*p*‐value	*t*/*X* ^2^
Gender			0.780	0.078
Male	26	27		
Female	9	8		
Age	60.31 ± 9.40	62.11 ± 8.44	0.402	0.843
Differentiation			0.799	0.065
Moderately	12	11		
Poorly	23	24		
Tumor site			0.196	3.257
Cardia	26	21		
Body	1	5		
Antrum	8	9		
Tumor size (diameter, cm)	7.98 ± 1.78	7.446 ± 1.394	0.165	1.404
Histology			0.586	1.069
Adenocarcinoma	30	28		
Mucinous adenocarcinoma	1	3		
Signet ring cell type	4	4		
Lauren classification			0.401	1.826
Intestinal type	1	1		
Diffuse type	12	7		
Hybrid type	22	27		
T pathological stage			0.303	1.061
T3	3	1		
T4	32	34		
N pathological stage			0.303	1.061
2	2	1		
3	33	34		
M stage			1	0
0	23	23		
1	12	12		
Clinical stage			0.799	0.065
III	23	24		
IV	12	11		

**FIGURE 1:**
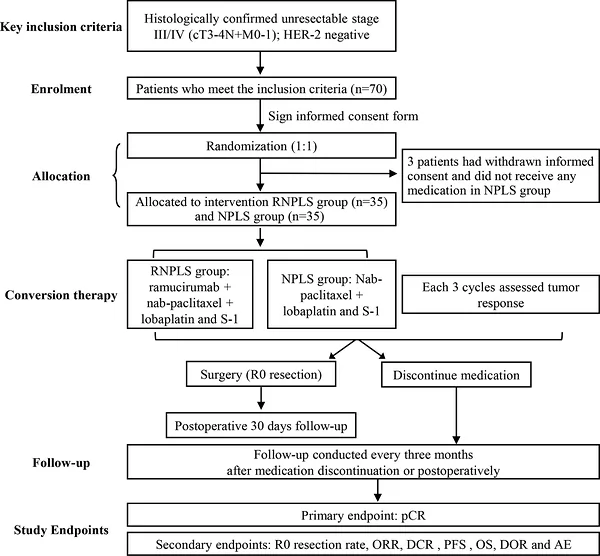
The flowchart of the conversion therapy cohort of RNPLS‐01 trial.

### 2.2 The Efficacy of the Patients After Conversion Therapy

Three patients in the NPLS group had withdrawn informed consent and did not receive any medication post‐randomization. The RNPLS group demonstrated significantly promising efficacy outcomes across multiple parameters compared to the NPLS group (Figure 2A, Table 2). For best overall response (BOR), neither group achieved complete response (CR). Stable disease (SD) occurred in nine RNPLS group patients versus 14 NPLS group patients. The RNPLS group showed significantly higher partial response rates (PR: 24 vs. 7) and fewer progressive disease rates (PD: 2 vs. 11) compared to the NPLS group, with statistical significance (*p* < 0.0001, Figure 2B, Table 2). Markedly promising outcomes were observed in the RNPLS group for both ORR (65.71% [23/35] vs. 25.71% [9/35], *p* = 0.002) and disease control rate (DCR: 94.29% [33/35] vs. 62.86% [22/35], *p* = 0.006, Table 2). In addition, the clinical benefit rate (CBR), defined as CR/PR/SD sustained ≥6 months, was significantly higher in the RNPLS group (94.29% vs. 65.63%, *p* = 0.003, Table 2). The representative pre‐ and post‐treatment CT images reflecting radiographic responses of the two groups are shown in Figure 3.

**FIGURE 2:**
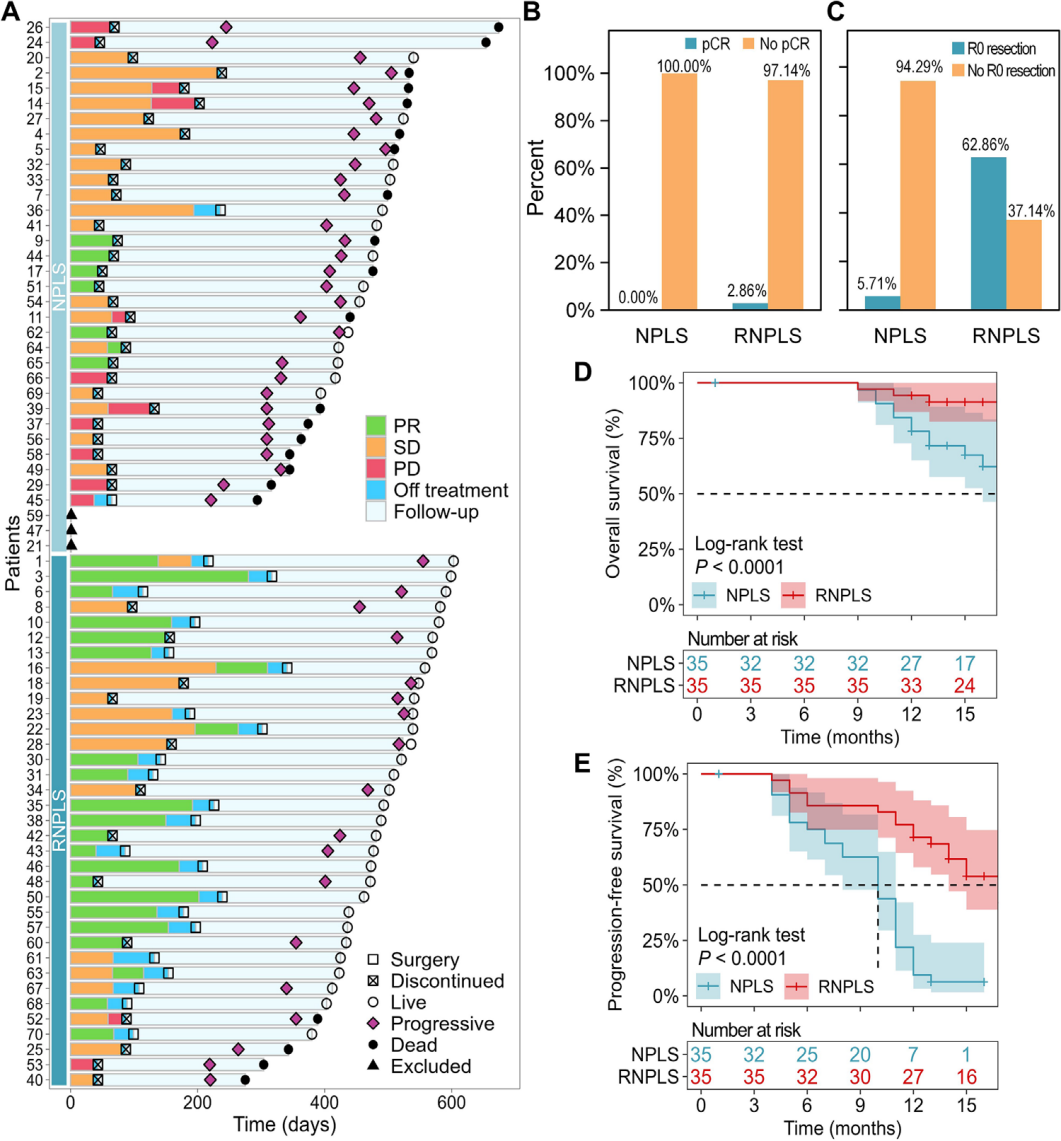
Several efficacy outcomes between two groups of the conversion therapy cohort of RNPLS‐01 trial. (A) Swimmer plot of time on conversion therapy. Each bar represents one participant. (B) Best overall response compared between the two groups. (C) pCR compared between the two groups. (D) Kaplan–Meier curves of progression‑free survival (PFS) in the RNPLS and NPLS groups. Number‑at‑risk tables are shown below the curve, and censoring events are marked with vertical ticks. Log‑rank test, *p* < 0.0001. (E) Kaplan–Meier curves of overall survival (OS) in the RNPLS and NPLS groups. Number‑at‑risk tables are shown below the curve, and censoring events are marked with vertical ticks. Log‑rank test, *p* < 0.0001.

**TABLE 2:** Comparison of efficacy outcomes between RNPLS group and NPLS group in conversion therapy cohort.

Efficacy outcomes	RNPLS group (*n* = 35)	NPLS group (*n* = 35)	*p*‐value
Best overall response(BOR), *n*			< 0.0001
Complete response (CR)	0	0	
Partial response (PR)	24	7	
Stable disease (SD)	9	14	
Progressive disease (PD)	2	11	
Objective response rate (ORR), %	23 (65.71%)	9 (25.71%)	0.002
Disease control rate (DCR), %	33 (94.29%)	22 (62.86%)	0.006
Clinical benefit rate (CBR), %	33 (94.29%)	21 (65.63%)	0.003
R0 resection rate (conversion therapy success rate)	22 (62.86%)	2 (5.71%)	< 0.0001
pCR			1.000
TRG 0	1	0	
TRG 1	3	0	
TRG 2	12	0	
TRG 3	5	2	
No TRG (no surgery)	14	33	
Duration of response (DOR), mean, months	7.49 ± 6.20	2.29 ± 5.32	0.0003
Median OS (95% CI)	Not reached	17 (16–not reached)	< 0.0001
Mean OS (95% CI)	18.31 ± 0.39 (17.55–19.07)	15.23 ± 0.49 (14.28–16.18)	< 0.0001
Median PFS (95% CI)	17 (14–not reached)	10 (8–11)	< 0.0001
Mean PFS (95% CI)	14.76 ± 0.82 (13.15–16.37)	9.31 ± 0.58 (8.19–10.44)	< 0.0001
6‐Month overall survival rate (%)	100% (35/35)	100% (32/32)	−
6‐Month progression‐free survival rate (%)	82.86% (29/35)	75.00% (24/32)	0.429
12‐Month overall survival rate (%)	94.29 % (33/35)	84.38% (27/32)	0.058
12‐Month progression‐free survival rate (%)	71.43% (25/35)	9.38% (3/32)	< 0.0001

**FIGURE 3:**
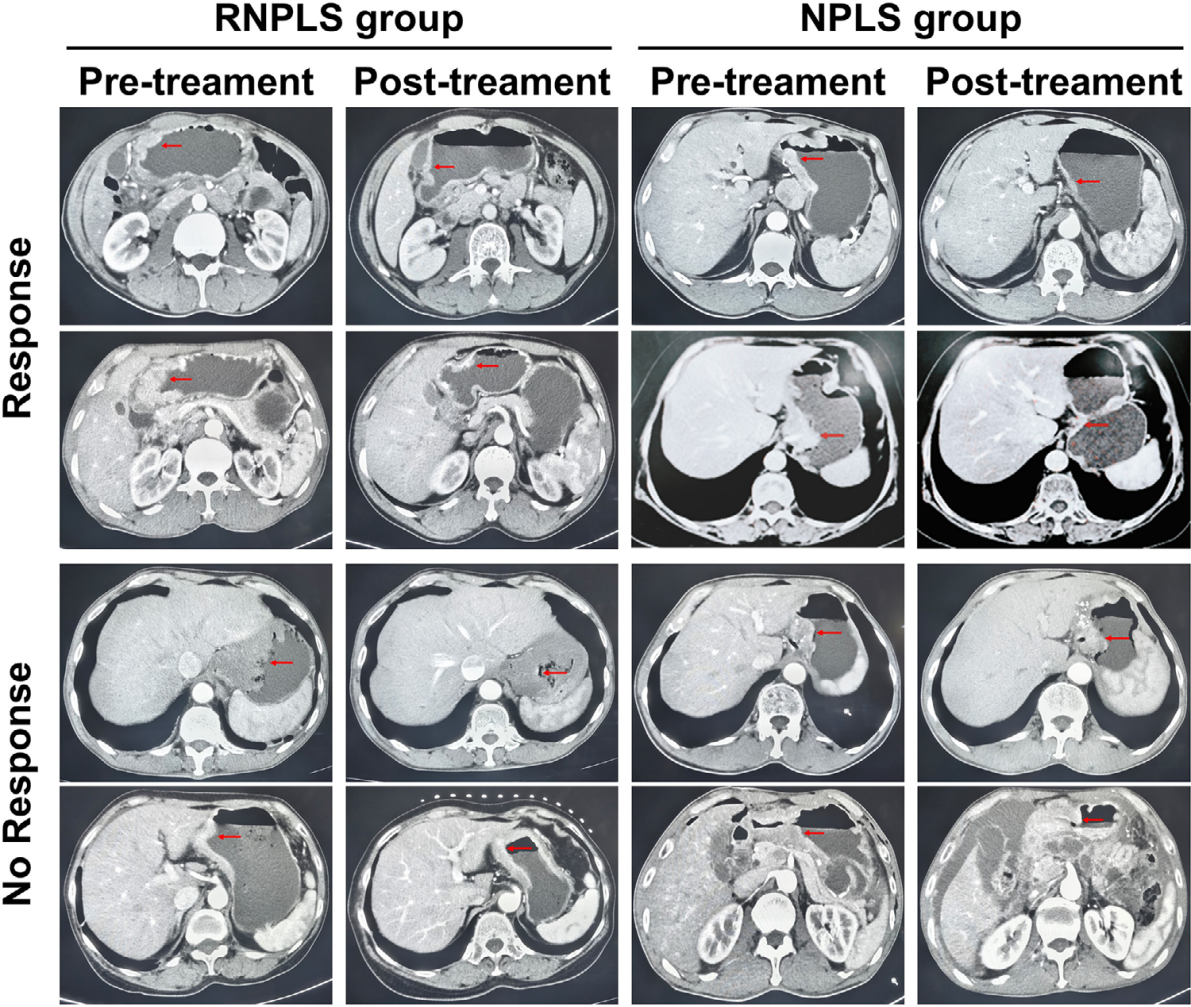
The representative CT images reflecting radiographic responses of patients pre‐ and post‐treatment in both groups. Response refers to PR or CR patients and no‐response refers to SD or PD patients.

Patients who achieved conversion success were treated with surgery. R0 resection rates (conversion therapy success rates) differed substantially between groups, with the RNPLS group showing promising R0 resection rates (62.86% [22/35] vs. 5.71% [2/35], *p* < 0.0001, Table 2). Nevertheless, this between‐group difference should be interpreted with caution given unblinded surgical assessment and lack of pre‐specified written conversion criteria, which may lead to potential investigator‐dependent selection bias for surgical indication. The RNPLS group had one patient achieved TRG 0, three achieved TRG 1, and 12 achieved TRG 2 versus no TRG 0–2 cases in the NPLS group. TRG 3 was observed in five patients in the RNPLS group and two patients in the NPLS group. In addition, 14 patients in the RNPLS group and 33 patients in the NPLS group lacked TRG assessment due to non‐surgical management. The pCR rate was 2.86% (1/35) in the RNPLS group and 0% (0/35) in the NPLS group, with no significant difference between groups (Fisher's exact test, *p* = 1.000; Table 2). In addition, a total of 22 patients in the RNPLS group and two patients in the NPLS group underwent surgery and were evaluated for pCR and TRG. Accordingly, 14 patients in the RNPLS group and 33 patients in the NPLS group did not receive surgery and lacked TRG assessment. The pCR rate was 4.55% (1/22) in the surgical subgroup of the RNPLS group and no pCR (0/2) was observed in surgical subgroup of the NPLS group. Of note, the non‐significant difference in pCR between two arms was mainly attributed to small sample size and inherently low pCR incidence among unresectable advanced GC receiving conversion therapy. All secondary efficacy indicators achieved statistically prominent improvement, which should be interpreted cautiously per the immature follow‐up data and single‐center exploratory design.

As of July 31, 2025 (ongoing follow‐up), median follow‐up was 9 months (range 5–15, 95% CI 8.74–10.46) for RNPLS group and 11 months (range 8–16, 95% CI 10.15–11.66) for NPLS group intention‐to‐treat populations. Median OS was not reached in the RNPLS group, indicating that survival data remain immature. Despite this, significantly prolonged PFS and favorable OS outcomes were observed in the RNPLS group. In addition, T the RNPLS group exhibited significantly prolonged duration of response (DOR: 7.49 ± 6.20 vs. 2.29 ± 5.32 months, *p* = 0.0003, Table 2).

There were three deaths in the RNPLS group and 17 deaths in the NPLS group. Survival analyses showed that the RNPLS group demonstrated promising OS (median OS: not reached by 19 months [95% CI: not reached] vs. 17 months [95% CI: 16–not reached]; mean OS: 18.31 ± 0.39 months [95% CI: 17.55–19.07] vs. 15.23 ± 0.49 months [95% CI: 14.28–16.18], *p* < 0.0001, Figure 2D, Table 2) and PFS (median PFS: 17 months [95% CI: 14–not reached] vs. 10 months [95% CI: 8–11]; mean PFS: 14.76 ± 0.82 months [95% CI: 13.15–16.37] vs. 9.31 ± 0.58 months [95% CI: 8.19–10.44], *p* < 0.0001, Figure 2E, Table 2) than NPLS group. Both groups achieved 100% 6‐month OS, with comparable 6‐month PFS (82.86% [29/35] vs. 75.00% [24/32], *p* = 0.428, Table 2). However, significant divergene emerged at 12 months, 12‐month PFS was 71.43% (25/35) for RNPLS group vs. 9.38% (3/32) for NPLS group (*p* < 0.0001), while 12‐month OS remained comparable (94.29% [33/35] vs. 84.38% [27/32], *p* = 0.058, Table 2).

Preliminary results demonstrate that the RNPLS regimen significantly improved tumor response, conversion resectability, and PFS. Given the short median follow‑up and immature OS data, the observed OS benefit should be interpreted with caution and requires confirmation with extended follow‑up.

### 2.3 The Safety and Tolerability of Enrolled Patients

Details of treatment‐related adverse events (TRAEs) during conversion therapy are summarized in Table 3. In the RNPLS group, 77.14% (27/35) of patients experienced at least one TRAE of any grade, comprising 71.43% (25/35) with grade 1–2 events and 5.71% (2/35) with grade 3 events. The NPLS group showed comparable rates, with 71.88% (23/32) of patients reporting any‐grade TRAEs, including 62.86% (22/35) grade 1–2 and 3.13% (1/32) grade 3 events. Statistical analysis revealed no significant differences between groups in overall TRAE incidence (*p* = 0.621), grade 1–2 TRAE events (*p* = 0.811), or grade 3 TRAE events (*p* = 0.609). Notably, neither group experienced surgical complications or treatment‐related mortality (grade 5 events) during the study period. The most frequent TRAEs demonstrated similar prevalence between groups (Figure 4): leukopenia (40.00% [14/35] vs. 40.63% [13/32], *p* = 0.958), neutropenia (14.29% vs. 15.63%, *p* = 0.878), decreased appetite (14.29% vs. 15.63%, *p* = 0.878), anemia (31.43% vs. 25.00%, *p* = 0.560), and malnutrition (25.71% vs. 25.00%, *p* = 0.946). Other comparable events included alopecia (31.43% vs. 18.75%, *p* = 0.234, Figure 4), thrombocytopenia (11.43% vs. 3.13%, *p* = 0.196, Figure 4), fatigue (22.86% vs. 12.50%, *p* = 0.269, Figure 4), gastrointestinal symptoms (14.29% vs. 21.88%, *p* = 0.418, Figure 4), and liver function abnormalities (11.43% vs. 9.38%, *p* = 0.784, Figure 4). Hypertension emerged as the sole statistically significant difference between groups, occurring in 25.71% (9/35) of RNPLS group patients versus 3.13% (1/32) of NPLS group patients (*p* = 0.010, Figure 4). All AEs were managed effectively through supportive care, with detailed documentation provided in Table S1.

**TABLE 3:** Comparison of treatment‐related adverse events (AEs) occurred between RNPLS group and NPLS group of enrolled patients.

Treatment‐related adverse events	RNPLS group (*n* = 35)	NPLS group (*n* = 32)^a^	*p*‐value	*t*/*X* ^2^
Any grades	27 (77.14%)	23 (71.88%)	0.621	0.245
Grade 1/2	25 (71.43%)	22 (62.86%)	0.811	0.057
Grade 3/4	2 (5.71%)	1 (3.13%)	0.609	0.262
Hypertension	9 (25.71%)	1 (3.13%)	0.010	6.718
Proteinuria	1 (2.86%)	1 (3.13%)	0.949	0.004
Fatigue	8 (22.86%)	4 (12.50%)	0.269	1.220
Gastrointestinal symptoms^b^	5 (14.29%)	7 (21.88%)	0.418	0.655
Bleeding/hemorrhage events^c^	4 (11.43%)	2 (6.25%)	0.458	0.550
Leukopenia	14 (40.00%)	13 (40.63%)	0.958	0.003
Neutropenia	5 (14.29%)	5 (15.63%)	0.878	0.024
Lymphocytopenia	5 (14.29%)	2 (6.25%)	0.283	1.154
Anemia	11 (31.43%)	8 (25.00%)	0.560	0.340
Thrombocytopenia	4 (11.43%)	1 (3.13%)	0.196	1.669
Decreased appetite	5 (14.29%)	5 (15.63%)	0.878	0.024
Malnutrition	9 (25.71%)	8 (25.00%)	0.946	0.005
Arterial/Venous thromboembolic event^d^	1 (2.86%)	0	0.335	0.928
Liver function damage	4 (11.43%)	3 (9.38%)	0.784	0.075
Alopecia	11 (31.43%)	6 (18.75%)	0.234	1.419
Palmar‐plantar erythrodysesthesia/rash	7 (20.00%)	8 (25.00%)	0.624	0.241

^a^ Three patients in the NPLS group had withdrawn informed consent and did not receive any medication post‐randomization.

^b^ Gastrointestinal symptoms include diarrhea and constipation.

^c^ Bleeding/hemorrhage events including nosebleeds or gum bleeding.

^d^ Arterial/venous thromboembolic event include myocardial infarction and cerebral infarction.

**FIGURE 4:**
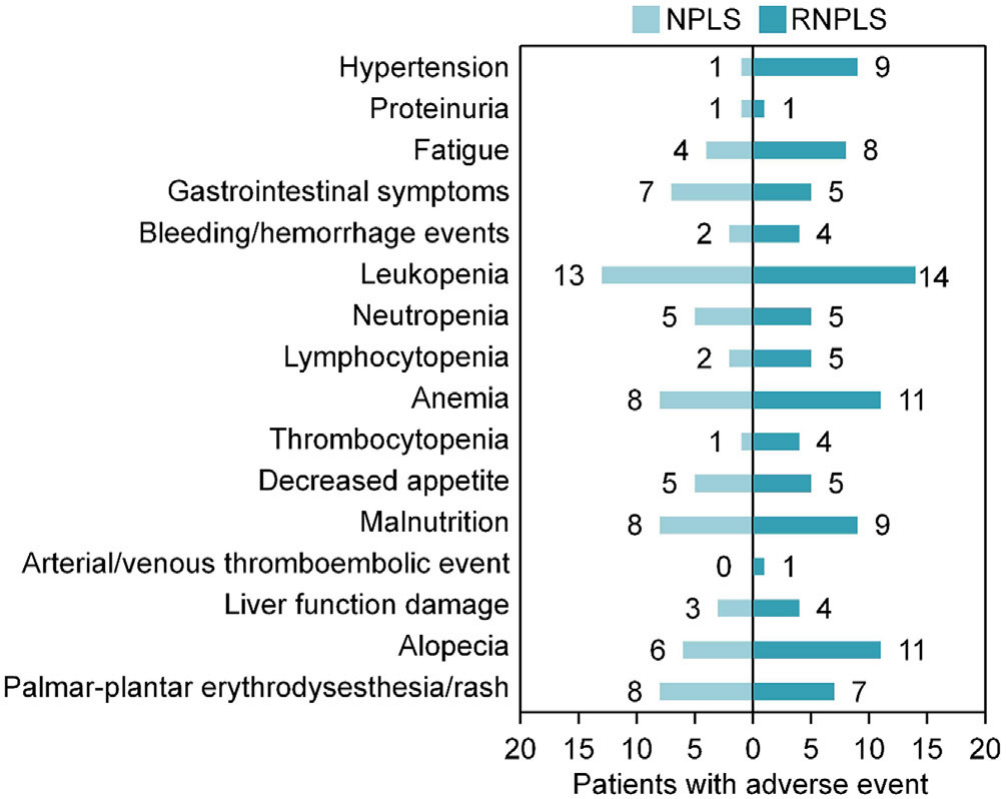
Treatment‐related adverse events in the two groups of the conversion therapy cohort of the RNPLS‐01 trial.

In addition, mean relative dose intensity (RDI) was 86.3%–89.1% for individual drugs in RNPLS group and 87.2%–90.4% in NPLS group. Dose reduction was conducted in 11.43% (4/35) of RNPLS patients and 9.38% (3/32) of NPLS patients; treatment cycle postponement happened in 8.57% (3/35) and 6.25% (2/32), respectively.

The safety profiles of both groups were generally comparable, with low rates of severe toxicities (grade ≥3 events) observed in either cohort. The low incidence of grade ≥3 TRAEs was closely associated with optimized baseline dosage, timely dose adjustment, standardized proactive supportive care, and inherent low‐toxicity features of nab‐paclitaxel and lobaplatin in Asian patients. These findings suggest that while hypertension requires particular attention in RNPLS‐treated patients, both treatment approaches demonstrate similar tolerability profiles during conversion therapy.

## 3 Discussion

The RNPLS‐01 trial provides preliminary evidence that the combination of ramucirumab with nab‐paclitaxel, lobaplatin, and S‐1 (RNPLS regimen) enhances therapeutic efficacy in conversion therapy for unresectable advanced GC. Recent global phase III trials have established the efficacy of immune‑chemotherapy and anti‑angiogenic regimens in advanced GC (ARMANI, KEYNOTE‑859, CheckMate‑649, ATTRACTION‑4, ORIENT‐16, GEMSTONE‐303, KEYNOTE‐811, RATIONALE‐305) [10, 11, 12, 13, 14, 15, 41]. The regimen achieved an ORR of 65.71% in the RNPLS group substantially exceeding historical benchmarks of 30%–45% for conventional platinum‐fluoropyrimidine regimens and aligning with the synergistic potential predicted by RAINBOW trial, where ramucirumab‐paclitaxel combinations improved OS [26, 27, 42]. Notably, this quadruplet therapy regimen achieved a notable 62.86% conversion success rate, enabling curative R0 resection in over half of patients—a crucial advancement given that complete resection remains the cornerstone of long‐term survival in unresectable advanced GC. These statistically significant secondary endpoint improvements suggest potential clinical benefit of RNPLS regimen. However, these findings should be interpreted cautiously considering single‐center design, limited sample size, and immature OS follow‐up data, pending validation in larger prospective trials.

The promising response metrics (ORR: 65.71% vs. 25.71%, *p* = 0.002; DCR: 94.29% vs. 62.86%, *p* = 0.006) support the hypothesized synergy between anti‐angiogenic therapy and cytotoxic agents. As a VEGFR‐2 inhibitor, ramucirumab disrupts tumor angiogenesis through VEGF signaling blockade while promoting vascular normalization and reducing tumor hypoxia‐mechanisms that may enhance chemotherapy penetration and mitigate hypoxia‐mediated resistance [43]. Additionally, preclinical evidence further suggests that anti‐angiogenic agents like ramucirumab could potentiate T‐cell infiltration and immunotherapy synergy [44]. Although not explored here, combining RNPLS with PD‐1 inhibitors (e.g., nivolumab) warrants investigation, as seen in ATTRACTION‐2 [45]. The albumin‐bound formulation of nab‐paclitaxel avoids solvent‐related toxicity (e.g., Cremophor‐induced hypersensitivity) and leverages SPARC protein binding in tumor stroma to improve intratumoral drug accumulation, further improving drug delivery [46]. Preclinical studies indicate that nab‐paclitaxel enhances tumor penetration by fivefold compared to solvent‐based paclitaxel, a critical factor in achieving deeper responses [47]. Moreover, lobaplatin, a third‐generation platinum agent, induces DNA cross‐linking, and S‐1 (a fluoropyrimidine) inhibits thymidylate synthase. This combination exploits complementary cytotoxic pathways, particularly effective in Asian populations likely contributed to the regimen's potency by targeting DNA repair and inducing cell cycle arrest by genetic polymorphisms affecting fluoropyrimidine metabolism [48, 49, 50, 51].

Despite the favorable conversion rate, the pathological complete response (pCR) rate was only 2.86% in the RNPLS group, with no pCR was observed in the control group. The between‐group difference in the primary endpoint pCR was not statistically significant (2.86% vs. 0%, *p* = 1.000), indicating that the trial failed to meet its predefined primary endpoint. Nevertheless, the RNPLS regimen substantially improved the R0 resection rate and survival outcomes, verifying its clinical value in conversion therapy for unresectable advanced GC. This discrepancy reflects a key property of anti‐angiogenic therapies. Such treatments can induce tumor necrosis and vascular normalization but are unable to eliminate residual malignant clones [52]. Histopathological examination of surgical specimens further confirmed this finding. Most patients who responded to treatment achieved tumor regression grade (TRG) 1–2 (partial regression) rather than TRG 0 (complete regression), which is consistent with previous studies demonstrating that ramucirumab mainly causes stromal necrosis instead of complete tumor eradication [53]. Two primary factors account for the negative result of the primary endpoint. First, the conversion cohort had an insufficient sample size that failed to meet the pre‐calculated requirement for pCR comparison, leading to inadequate statistical power. Second, the anti‐angiogenic effect of ramucirumab predominantly triggers tumor stroma degeneration and partial tumor regression (TRG 1–2), rather than complete pathological clearance of tumor cells (TRG 0/pCR). In addition, pCR is inherently a rare outcome among patients with unresectable advanced GC receiving conversion therapy in real clinical practice. Notably, owing to non‐blinded resectability evaluation and absence of predefined standardized conversion surgery criteria, investigator assessment bias cannot be fully excluded as a contributing factor for the large intergroup difference of conversion success. Further trials with pre‐established explicit conversion criteria and blinded central imaging review are required to validate our present conversion outcomes.

The OS and PFS advantage and promising 12‐month PFS rate highlights the durability of response in the RNPLS group, suggesting that conversion therapy not only enables surgery but also sustained tumor suppression. These findings align with RAINBOW subgroup analyses, where responders to ramucirumab‐paclitaxel exhibited prolonged survival [26, 27, 42]. Of note, this promising between‐group difference is exploratory and limited by multiple inherent trial design restrictions, which precludes definitive clinical inference and widespread generalization. Notably, the magnitude of intergroup differences in R0 resection rate, PFS and 12‐month PFS in our study is considerably larger than most prior ramucirumab‐based clinical trials. Several key factors collectively account for this prominent treatment effect. First, unlike previous trials combining ramucirumab with single‐agent paclitaxel, RNPLS is a quadruple combination integrating anti‐angiogenic agent plus three cytotoxic drugs with complementary anti‐tumor mechanisms. Second, all participants were untreated first‐line unresectable GC patients, while nearly all historical ramucirumab studies enrolled second‐line pretreated tumors with inherent chemoresistance. Third, lobaplatin and S‐1 exhibit superior anti‐tumor activity in East Asian GC population compared with conventional Western platinum‐fluoropyrimidine regimens. Nevertheless, owing to single‐center design and limited sample size, such prominent treatment effect cannot be generalized to broader patient populations across different institutions and ethnic groups.

In the Phase 3 RAINBOW trial involving advanced cancer patients, the combination of paclitaxel and ramucirumab demonstrated an 80% DCR (95% CI 75–84) with median PFS of 4.4 months (95% CI 4.2–5.3) in second‐line treatment [26]. In the RAMIRIS trial of AIO in 2022, a very similar patient population (100% of patients received platinum plus fluoropyrimidine pretreatment, 68% of patients received taxane drug pretreatment) received second‐line ramucirumab plus paclitaxel or FOLFIRI treatment [54]. The median OS of RAMIRIS is 7.6 months. In the Western population of the RAINBOW trial, patients who received platinum plus fluoropyrimidine pre‐treatment but did not receive taxane pre‐treatment had a median OS of 8.6 months, which was 2 months shorter than the RAP trial, which mainly treated patients with taxane [26, 55]. Compared with the OS found in the RAIN study, the OS of the group treated with ramucirumab combined with S‐1 and oxaliplatin, as well as placebo combined with S‐1 and oxaliplatin, was relatively longer, which is consistent with previous studies conducted in East Asia [56]. In addition, as is well known, paclitaxel has anti angiogenic activity [57], therefore, its potential synergistic effect with ramucirumab may help improve the prognosis of second‐line treatment for advanced GC.

Safety analyses revealed no novel safety signals related to ramucirumab in current trial. The safety of ramucirumab in current studies is controllable and consistent with previously published studies on the treatment of metastatic GC or gastroesophageal junction adenocarcinoma patients with ramucirumab [58] and the RAINBOW study of second‐line paclitaxel plus ramucirumab [26]. The RNPLS regimen maintained manageable toxicity, with only 5.71% experiencing grade ≥3 AEs. The incidence of grade ≥3 AEs was lower than previously reported for taxane‑ and platinum‑based regimens. Although RNPLS is a four‐drug combination, the observed low rate of grade ≥3 AEs were supported by collected RDI, dose reduction, and treatment delay data. Strict pre‐set optimized dosage, timely individual dose modification upon emerging toxicity, routine prophylactic supportive care and favorable safety profiles of lobaplatin and nab‐paclitaxel in East Asian population jointly account for the mild toxicity profile. Hypertension, a class effect of VEGFR‐2 inhibitors, was more frequent in the RNPLS group (25.71% vs. 3.13%, *p* = 0.010). This aligns with RAINBOW (25.1% incidence) and was effectively controlled with standard antihypertensives [26]. Notably, the incidence of severe neutropenia (14.29%) was lower than that in the B‐RAX trial (38.4% with ramucirumab + nab‐paclitaxel), likely due to dose optimization of nab‐paclitaxel (100 mg/m^2^ vs. 125–150 mg/m^2^ in other studies) [59]. The comparable AE rates between RNPLS and NPLS groups (*p* = 0.621 for overall TRAEs) challenge the assumption that quadruplet therapy inherently increases toxicity. This tolerability may stem from nab‐paclitaxel's reduced solvent‐related toxicity and lobaplatin's favorable safety profile in Asian populations [47, 48, 49, 50, 51, 60]. Of note, these favorable safety outcomes were derived from single‐center limited sample, and future larger trials are required to validate the safety of this quadruple regimen. In follow‐up prospective research, combining PD‐1 inhibitor with RNPLS regimen can be further explored to integrate anti‐angiogenic and immunotherapeutic advantages, aiming to further boost conversion success rate referring to current mainstream chemo‐immunotherapy paradigm.

Our study has several limitations. First, the conversion cohort was designed as an exploratory subgroup of the overall RNPLS‐01 trial, resulting in insufficient sample size and inadequate statistical power for the predefined primary endpoint of pCR despite meaningful improvements in secondary endpoints including R0 resection and survival outcomes. The observed positive secondary results cannot fully compensate primary endpoint failure, and all promising efficacy findings require further verification in adequately powered, prospectively randomized controlled trials. Second, selecting pCR as primary endpoint is suboptimal for conversion therapy of unresectable advanced GC given its naturally low incidence in this patient population, leading to the statistical failure of primary endpoint even when the regimen achieves prominent conversion resection benefit. In contemporary conversion therapy trials, R0 resection rate, PFS, and OS are more appropriate primary endpoints. Notably, the RNPLS regimen demonstrated improvements in all these clinically meaningful endpoints, confirming its clinical value. Thirdly, substantial missing pathological data may introduce selection bias. A considerable proportion of patients did not undergo surgery and therefore lacked pCR and TRG evaluation, especially in the NPLS group. Since only patients with favorable tumor response were eligible for resection, this may lead to overestimation of pathological response. The denominator for pCR and TRG was strictly defined as patients who underwent surgery, and results were reported accordingly. This limitation should be considered when interpreting pathological response endpoints. Fourth, although the R0 resection rate was significantly higher in the RNPLS group, surgical resectability assessment was performed in an unblinded fashion without pre‐specified standardized written conversion surgery criteria. Investigator discretionary bias for surgical indication cannot be completely excluded and may partially contribute to the observed large discrepancy in R0 resection rate between two groups. Therefore, the high conversion success rate observed in RNPLS arm cannot be entirely attributed to the anti‐tumor activity of the RNPLS regimen. Future conversion therapy trials should establish uniform written conversion criteria in advance and adopt blinded central radiology review to avoid such assessment bias. Fifth, the choice of the NPLS control regimen may limit the generalizability of the findings. The combination of nab‑paclitaxel, lobaplatin, and S‑1 is not a standard or widely used regimen in international clinical practice, which may reduce the external validity of the study. The NPLS regimen was adopted primarily based on its favorable toxicity and efficacy profile in Asian patients, and the results should be interpreted accordingly. Sixth, the unexpectedly low high‐grade AE rate was observed in a small single‐center cohort; centralized real‐world multi‐center data are needed to further confirm the toxicity profile of the four‐agent RNPLS regimen. Finally, this is an exploratory, single‐center, phase II trial with a small sample size and relatively short follow‑up. The results are inadequate to modify current clinical practice. Caution is warranted in interpreting the observed survival benefits due to potential biases and immature data.

In conclusion, our exploratory single‐center data identifies potential clinical benefits of RNPLS regimen. The RNPLS regimen yields promising preliminary improvements in tumor response, conversion resectability, and PFS in patients with unresectable advanced GC, with manageable toxicity. Given the small sample size, short follow‑up, unblinded surgical assessment and potential biases, the present exploratory study was statistically negative for primary endpoint pCR, precluding definitive clinical recommendations, while secondary endpoint results showed promising efficacy signals of RNPLS for conversion therapy, which needs further large‐scale, multicenter, prospectively randomized controlled trials validation.

## 4 Methods and Analysis

This prospective single‐center, two‐arm randomized controlled trial (RNPLS‐01) was approved by the Ethics Committee of the First Affiliated Hospital (Xijing Hospital) of the Fourth Military Medical University (KY20232220‐F‐1). The RNPLS‐01 trial aimed to evaluate the efficacy and safety of ramucirumab combined with nab‐paclitaxel, lobaplatin, and S‐1 in neoadjuvant and conversion therapy for patients with gastric or gastroesophageal junction adenocarcinoma. This trial complies with the Helsinki Declaration, International Conference on Harmonisation Good Clinical Practice guidelines, and relevant local regulations. The trial protocol detailing study design, inclusion/exclusion criteria, dosage regimens, sample size calculation, blinding methods, efficacy assessment parameters, data collection procedures, observation indicators, follow‐up protocols, analytical dataset definitions, data management, and statistical methods has been previously published [61]. Written informed consent was obtained from all participants along with their guardians, primary caregivers, and research assistants. All authors had access to the study data and had reviewed and approved the final manuscript.

For the conversion therapy cohort of the RNPLS‐01 trial, the main inclusion criteria were (1) age 18–75 years with Eastern Cooperative Oncology Group (ECOG) performance status ≤1; (2) histologically confirmed stage III/IV (cT3‐4N+M0‐1) unresectable advanced GC (HER‐2 negative); (3) no prior anticancer therapies; (4) adequate organ function within 1 week pre‐treatment: hemoglobin >80 g/L, absolute neutrophil count >1.5 × 10^9^/L, platelet count >100 × 10^9^/L (without recent transfusions), total bilirubin ≤1.5 × upper limit of normal (ULN), alanine aminotransferase/aspartate aminotransferase ≤2.5 × ULN (≤5 × ULN with liver metastases), serum creatinine ≤1.5 × ULN or creatinine clearance ≥50 mL/min, urinary protein <2+, and activated partial thromboplastin time/international normalized ratio ≤1.5 × ULN; and (5) expected survival >6 months.

Participants were recruited from Xijing Hospital, Fourth Military Medical University, with a predetermined conversion therapy cohort sample size of 70 patients. Between January 2023 and May 2024, all eligible patients were randomized (1:1) to either the RNPLS group (ramucirumab plus nab‐paclitaxel, lobaplatin, and S‐1) or NPLS group (control: nab‐paclitaxel, lobaplatin, and S‐1). The RNPLS group received intravenous ramucirumab (8 mg/kg), lobaplatin (30 mg/m^2^), and nab‐paclitaxel (100 mg/m^2^) on Day 1 of each 21‐day cycle, with oral S‐1 administered for 2 weeks followed by 1 week off. On the first day of each 21‐day cycle, patients in the control group were intravenously injected with lopaplatin (30 mg/m^2^) and nab paclitaxel (100 mg/m^2^), while taking S‐1 orally for 2 weeks and stopping for 1 week. S‐1 dosage was body surface area‐adjusted: <1.25 m^2^: 40 mg; 1.25–1.5 m^2^: 50 mg; >1.5 m^2^: 60 mg. Conversion therapy continued until radiological progression (RECIST 1.1), intolerable toxicity, consent withdrawal, or achievement of resectability (CR/PR per RECIST 1.1) prompting curative surgery. All candidates for potential conversion surgery were comprehensively evaluated by institutional multidisciplinary team (MDT) consisting of senior gastric surgeons, abdominal radiologists, and medical oncologists for both study arms according to unified clinical practice standard based on RECIST 1.1 radiological response. Operation eligibility was determined when imaging demonstrated significant shrinkage of primary tumor, regression of regional lymph node metastasis, and disappearance of locally invasive extra‐gastric lesions. Notably, formal written standardized conversion surgical criteria were not pre‐defined in the trial protocol, and investigators remained unblinded to treatment allocation during resectability assessment, which may introduce evaluation bias. The trial was registered at ClinicalTrials.gov (NCT06169410; registered December 5, 2023).

The primary endpoint was pCR, assessed through postoperative pathological tumor regression grading (TRG), as previously described [62]. Secondary endpoints included R0 resection rate, ORR, DCR, PFS, OS, duration of response (DOR), and AEs. ORR was defined as the proportion of patients achieving CR or partial response (PR) by RECIST 1.1. DCR included CR, PR, and SD. PFS spanned from enrollment to progression/death, while OS measured time from enrollment to death from any cause. DOR represented time from initial response to progression/death. Furthermore, patients who successfully underwent conversion therapy received surgical treatment. The conversion therapy success rate was defined as the proportion of patients who undergo surgical treatment after conversion therapy, including those who undergo R0 resection.

### 4.1 Safety Assessments

Safety was evaluated according to NCI CTCAE version 5.0. All AEs were monitored continuously throughout treatment and documented at every visit. Dose intensity, dose reductions, treatment delays, and supportive care interventions (including granulocyte colony‑stimulating factor [G‑CSF], antiemetics, and antihypertensive agents) were recorded in detail for all patients.

### 4.2 Dose Modifications

RDI, frequency of dose reduction and cycle delay were prospectively documented throughout treatment. Predefined optimized initial doses were used for all four agents. Prophylactic G‐CSF, antiemetics, and symptomatic antihypertensive treatment were routinely available as supportive care. Dose reduction was implemented for persistent grade ≥3 hematological or non‐hematological toxicities; treatment was delayed for ≥7 days when severe unresolved AEs occurred.

### 4.3 Sample Size Calculation

The sample size was calculated based on the primary endpoint, pCR rate. According to previous literature, the pCR rate of neoadjuvant or conversion chemotherapy for advanced GC was approximately 4.8%–10%, and the pCR rate of chemotherapy combined with targeted therapy was approximately 20%. We assumed a pCR rate of 10% in the control group (NPLS) and 20% in the experimental group (RNPLS). A one‐sided significance level of 0.025 and a statistical power of 80% were set. Using PASS 11.0 software, the target sample size was estimated to be 58 patients per group, 116 in total. With a 20% censoring rate, the total number of enrolled patients was 140. This trial consisted of two cohorts: a neoadjuvant cohort and a conversion‑therapy cohort. Seventy patients were enrolled in the conversion‑therapy cohort, with 35 patients each in the RNPLS and NPLS groups. The sample size calculation was originally performed for the entire RNPLS‐01 trial containing both neoadjuvant and conversion cohorts. Therefore, the independent conversion therapy cohort with only 70 patients was pre‐specified as an exploratory subgroup and statistically underpowered for primary endpoint pCR comparison from trial design.

### 4.4 Randomization, Allocation Concealment, and Stratification

This was a single‐center, randomized, controlled trial. Eligible patients were randomly assigned in a 1:1 ratio to the RNPLS group or the NPLS group. Randomization was performed using a computer‐generated random sequence created with SAS 9.4 software. Allocation concealment was implemented using sequentially numbered, sealed, opaque envelopes prepared by an independent statistician who was not involved in patient enrollment or treatment assignment. Investigators, outcome assessors, and statisticians were blinded to group allocation until all data analyses were completed. No stratification factors were applied in this trial.

### 4.5 Follow‐Up Protocol

For efficacy evaluation, patients underwent tri‐monthly imaging (contrast‐enhanced CT/MRI of chest/abdomen/pelvis) during treatment. Post‐treatment follow‐ups occurred every 3 months for years 1–2, then every 6 months from years 3–5 until death or 5‐year survival. Safety monitoring included 30‐day postoperative complication assessments for surgically treated patients, concluding 90 days post‐treatment discontinuation. All AEs were meticulously recorded with detailed documentation of onset, severity, management, and resolution to NCI CTCAE v5.0 grade ≤1 or complete recovery.

### 4.6 Statistical Analysis

Statistical analyses were performed using SAS version 9.4. The safety and efficacy analyses included all patients who received at least one dose of study treatment. Continuous variables were summarized as mean ± standard deviation or median (range), while categorical data were expressed as frequency and percentage. Intergroup comparisons for categorical variables utilized the chi‐square test. The primary endpoint (pCR) was compared between groups using Fisher's exact test for the 2×2 contingency table (pCR vs. non‑pCR), consistent with the sample size calculation based on pCR rate in the study protocol. In addition, pCR and TRG were also assessed in patients who underwent surgical resection after conversion therapy. In this analytical context, the denominator for pCR and TRG analyses was the number of patients with surgery. Patients without surgery were defined as having no TRG assessment and were excluded from pathological response evaluation. All statistical tests were two‐sided, with significance defined as *p* ≤0.05 and 95% confidence intervals. Survival curves were generated using the Kaplan–Meier method. For AE analyses, frequency and incidence rates were calculated. The cumulative incidence of serious adverse events (SAEs) was specifically quantified. In assessing AE severity, for recurrent instances of the same AE in a patient, only the most severe occurrence was included in the analysis; for multiple distinct AEs in a single patient, the highest grade AE were prioritized for inclusion.

## Author Contributions

J.W. and J.J.Y. conceived and designed the study. J.W., W.W., and X.H.L. wrote and reviewed the manuscript. G.H.X., S.S.L., L.X.J., and S.W. performed the trial. C.S.P., Y.X.M., X.L., and H.D.H. participated in patients’ follow‐up and H.L.L. participated in data management and statistical analysis. All authors read and approved the final version of the manuscript.

## Funding

This work is supported by grants from the Xijing Hospital 2024 Medical Personnel Training to Promote Clinical Special General Projects (Grant Nos. XJZT24LY32 and XJZT24LY34)—administered by Xijing Hospital of Digestive Diseases, Fourth Military Medical University, Xi'an, China., Key Research and Development Project of Shaanxi Province (Grant No. 2022ZDLSF03‐04)—administered by Department of Science and Technology of Shaanxi Province, People's Government of Shaanxi Province, Xi'an, China., Air Force Medical University clinical research program (Grant No. 2022LC2212)—administered by Air Force Medical University (formerly Fourth Military Medical University), Xi'an, China., and the Innovative exploratory project of the State Key Laboratory of Tumor Biology (Grant No. CBSKL2022ZZ41)—administered by State Key Laboratory of Tumor Biology, Fourth Military Medical University, Xi'an, China..

## Ethics Statement

Ethics approval has been obtained from the Ethics Committee at the First Affiliated Hospital (Xijing Hospital) of Air force Military Medical University (KY20232220‐F‐1). This trial was registered at the ClinicalTrials.gov: NCT06169410 (registration date: December 5, 2023). Written informed consent was obtained from all participants.

## Conflicts of Interest

The authors declare no conflicts of interest.

## Supporting information




**Supporting File 1**: mco270958‐sup‐0001‐tableS1.docx.

## Data Availability

The datasets generated and analyzed during the present study are available from the corresponding author on reasonable request.
