# Supplementary material for: Efficacy and Safety of ramucirumab Combined With Nab‐paclitaxel, Lobaplatin, and S‐1 in Conversion Therapy for Unresectable Advanced Gastric Cancer: Preliminary Results of Conversion Therapy Cohort of RNPLS‐01 Trial
Source: MedComm (2020). 2026 Nov 3;7(11):e70958. doi: 10.1002/mco2.70958 (PMC13732106; doi:10.1002/mco2.70958)
Supplement: Supplementary file 1 [file MCO2-7-e70958-s001.docx]

**Supplementary Table 1.** The patient's TRAE and treatment.

| Case | Gender | Age | Group | TRAE | Grade | Treatment | Outcome |
| --- | --- | --- | --- | --- | --- | --- | --- |
| 1 | M | 53 | RNPLS | Gastrointestinal symptoms, Anemia, Malnutrition, Lymphocytopenia | 1-2 | Supplementation | Recovery |
| 2 | M | 35 | NPLS | Hypertension, Proteinuria, Hemorrhage, Leukopenia, Alopecia | 3 | Pharmacotherapy | Recovery |
| 3 | F | 64 | RNPLS | Proteinuria, Fatigue, Lymphocytopenia, Decreased appetite, Malnutrition, Alopecia | 1-2 | Pharmacotherapy | Recovery |
| 4 | F | 69 | NPLS | Gastrointestinal symptoms, Leukopenia, Malnutrition | 1-2 | Pharmacotherapy | Recovery |
| 7 | M | 59 | NPLS | Fatigue, Anemia, Liver function damage, PPE/rash | 1-2 | Discontinue | Recovery |
| 8 | F | 63 | RNPLS | Hypertension, Fatigue, Neutropenia, Lymphocytopenia, PPE/rash | 1-2 | Pharmacotherapy | Recovery |
| 9 | M | 53 | NPLS | Fatigue, Gastrointestinal symptoms, Malnutrition, Liver function damage | 1-2 | Pharmacotherapy | Recovery |
| 10 | M | 71 | RNPLS | Decreased appetite, Alopecia, Liver function damage | 1-2 | Pharmacotherapy | Recovery |
| 11 | M | 66 | NPLS | Neutropenia, Anemia, Decreased appetite, Alopecia | 1-2 | Pharmacotherapy | Recovery |
| 12 | M | 70 | RNPLS | Gastrointestinal symptoms, Alopecia | 1-2 | Observation | Recovery |
| 13 | M | 61 | RNPLS | Leukopenia, Neutropenia, Anemia | 1-2 | Supplementation | Recovery |
| 14 | F | 56 | NPLS | Gastrointestinal symptoms, Anemia, Decreased appetite, Malnutrition, PPE/rash | 1-2 | Pharmacotherapy | Recovery |
| 15 | F | 45 | NPLS | Gastrointestinal symptoms, Malnutrition, Alopecia, PPE/rash | 1-2 | Pharmacotherapy | Recovery |
| 16 | M | 55 | RNPLS | Anemia, Decreased appetite, Malnutrition, Arterial/Venous thromboembolic event | 1-2 | Pharmacotherapy | Recovery |
| 17 | M | 64 | NPLS | Leukopenia, Alopecia, Anemia, Malnutrition, Gastrointestinal symptoms | 1-2 | Pharmacotherapy | Recovery |
| 18 | M | 64 | RNPLS | Fatigue, Alopecia | 1-2 | Observation | Recovery |
| 19 | M | 64 | RNPLS | Fatigue, Leukopenia | 1-2 | Pharmacotherapy | Recovery |
| 20 | M | 70 | NPLS | Leukopenia, Thrombocytopenia, Liver function damage, Alopecia | 1-2 | Pharmacotherapy | Recovery |
| 22 | M | 72 | RNPLS | Leukopenia, Neutropenia, Liver function damage, Alopecia, PPE/rash | 1-2 | Pharmacotherapy | Recovery |
| 23 | M | 61 | RNPLS | Leukopenia, Thrombocytopenia, Alopecia | 1-2 | Pharmacotherapy | Recovery |
| 25 | M | 41 | RNPLS | Gastrointestinal symptoms, Bleeding/hemorrhage events, Lymphocytopenia | 1-2 | Pharmacotherapy | Recovery |
| 27 | M | 47 | NPLS | Bleeding/hemorrhage events, Leukopenia, Neutropenia, Decreased appetite | 1-2 | Pharmacotherapy | Recovery |
| 28 | M | 69 | RNPLS | Bleeding/hemorrhage events, Leukopenia, Liver function damage, PPE/rash | 1-2 | Pharmacotherapy | Recovery |
| 30 | M | 52 | RNPLS | Leukopenia, Neutropenia, Malnutrition, PPE/rash | 1-2 | Supplementation | Recovery |
| 32 | M | 67 | NPLS | Leukopenia, Neutropenia, Decreased appetite, Malnutrition, PPE/rash | 1-2 | Supplementation | Recovery |
| 33 | M | 64 | NPLS | Leukopenia, Anemia, Alopecia | 1-2 | Supplementation | Recovery |
| 34 | M | 62 | RNPLS | Gastrointestinal symptoms, Anemia, Decreased appetite, Malnutrition, Alopecia | 1-2 | Pharmacotherapy | Recovery |
| 35 | M | 68 | RNPLS | Bleeding/hemorrhage events, Anemia, Malnutrition, Liver function damage | 3 | Pharmacotherapy | Recovery |
| 36 | M | 58 | NPLS | Fatigue | 1-2 | Observation | Recovery |
| 38 | M | 57 | RNPLS | Hypertension, Leukopenia, Thrombocytopenia | 1-2 | Pharmacotherapy | Recovery |
| 39 | M | 65 | NPLS | Lymphocytopenia, Malnutrition | 1-2 | Supplementation | Recovery |
| 40 | F | 57 | NPLS | Fatigue, Anemia, Thrombocytopenia, PPE/rash | 1-2 | Pharmacotherapy | Recovery |
| 41 | M | 72 | NPLS | PPE/rash | 1-2 | Observation | Recovery |
| 42 | M | 60 | RNPLS | Hypertension, Fatigue, Leukopenia | 1-2 | Pharmacotherapy | Recovery |
| 44 | M | 64 | NPLS | Fatigue, Leukopenia, Anemia, Decreased appetite | 1-2 | Pharmacotherapy | Recovery |
| 46 | M | 61 | RNPLS | Hypertension, Leukopenia, Anemia, PPE/rash | 1-2 | Pharmacotherapy | Recovery |
| 48 | F | 74 | RNPLS | Hypertension | 1-2 | Pharmacotherapy | Recovery |
| 49 | M | 66 | NPLS | Leukopenia | 1-2 | Pharmacotherapy | Recovery |
| 50 | M | 54 | RNPLS | Hypertension, Fatigue, Leukopenia, Anemia, Alopecia | 1-2 | Pharmacotherapy | Recovery |
| 51 | M | 56 | NPLS | Leukopenia, Malnutrition | 1-2 | Supplementation | Recovery |
| 52 | M | 65 | RNPLS | Hypertension, Bleeding/hemorrhage events, Leukopenia, Anemia, Malnutrition, Alopecia | 3 | Pharmacotherapy | Recovery |
| 56 | M | 70 | NPLS | Neutropenia | 1-2 | Pharmacotherapy | Recovery |
| 57 | F | 55 | RNPLS | Leukopenia, Anemia, Decreased appetite, Malnutrition | 1-2 | Pharmacotherapy | Recovery |
| 60 | M | 57 | RNPLS | Hypertension, Leukopenia | 1-2 | Pharmacotherapy | Recovery |
| 61 | M | 62 | RNPLS | Thrombocytopenia, Malnutrition, Alopecia, PPE/rash | 1-2 | Supplementation | Recovery |
| 62 | M | 60 | NPLS | Leukopenia | 1-2 | Pharmacotherapy | Recovery |
| 63 | F | 75 | RNPLS | Hypertension, Fatigue, Leukopenia, Neutropenia, Lymphocytopenia, Anemia, Alopecia | 1-2 | Pharmacotherapy | Recovery |
| 64 | F | 67 | NPLS | Gastrointestinal symptoms, Leukopenia, Neutropenia, Lymphocytopenia, PPE/rash | 1-2 | Pharmacotherapy | Recovery |
| 65 | M | 62 | NPLS | Gastrointestinal symptoms, Anemia, PPE/rash | 1-2 | Pharmacotherapy | Recovery |
| 69 | M | 67 | NPLS | Anemia, PPE/rash | 1-2 | Pharmacotherapy | Recovery |
